# Prediction of quality-adjusted life years (QALYs) after bariatric surgery using regularized linear regression models: results from a Swedish nationwide quality register

**DOI:** 10.1007/s11695-023-06685-1

**Published:** 2023-06-15

**Authors:** Sun Sun, Erik Stenberg, Lars Lindholm, Klas-Göran Salén, Karl A. Franklin, Nan Luo, Yang Cao

**Affiliations:** 1 Department of Epidemiology and Global Health, Umeå University, 901 87 Umeå, Sweden; 2 Department of Surgery, Faculty of Medicine and Health, Örebro University, 701 85 Örebro, Sweden; 3 Department of Surgical and Perioperative Sciences, Surgery, Umeå University, Umeå, Sweden; 4 Saw Swee Hock School of Public Health, National University of Singapore, Singapore, Singapore; 5 Clinical Epidemiology and Biostatistics, School of Medical Sciences, Faculty of Medicine and Health, Örebro University, 701 82 Örebro, Sweden; 6 Unit of Integrative Epidemiology, Institute of Environmental Medicine, Karolinska Institutet, 171 77 Stockholm, Sweden

**Keywords:** Bariatric surgery, Quality-adjusted life years, Prediction, Real-world data, SF-6D

## Abstract

**Purpose:**

To investigate whether the quality-adjusted life years (QALYs) of the patients who underwent bariatric surgery could be predicted using their baseline information.

**Materials and Methods:**

All patients who received bariatric surgery in Sweden between January 1, 2011 and March 31, 2019 were obtained from the Scandinavian Obesity Surgery Registry (SOReg). Baseline information included patients’ sociodemographic characteristics, details regarding the procedure, and postsurgical conditions. QALYs were assessed by the SF-6D at follow-up years 1 and 2. The general and regularized linear regression models were used to predict postoperative QALYs.

**Results:**

All regression models demonstrated satisfactory and comparable performance in predicting QALYs at follow-up year 1, with R^2^ and relative root mean squared error (RRMSE) values of about 0.57 and 9.6%, respectively. The performance of the general linear regression model increased with the number of variables; however, the improvement was ignorable when the number of variables was more than 30 and 50 for follow-up years 1 and 2, respectively. Although minor L1 and L2 regularization provided better prediction ability, the improvement was negligible when the number of variables was more than 20. All the models showed poorer performance for predicting QALYs at follow-up year 2.

**Conclusions:**

Patient characteristics before bariatric surgery including health related quality of life, age, sex, BMI, postoperative complications within six weeks, and smoking status, may be adequate in predicting their postoperative QALYs after one year. Understanding these factors can help identify individuals who require more personalized and intensive support before, during, and after surgery.

**Graphical Abstract:**

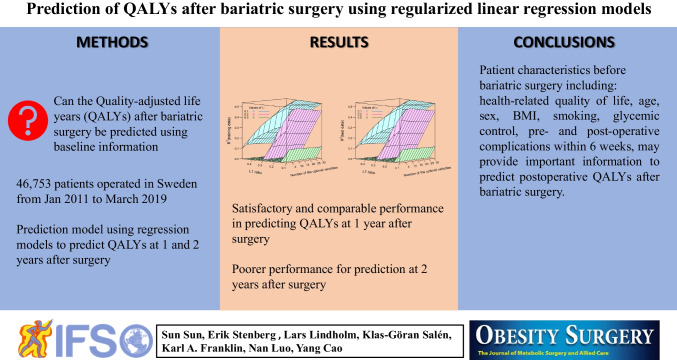

**Supplementary Information:**

The online version contains supplementary material available at 10.1007/s11695-023-06685-1.

## Introduction

Globally, the obesity rate is the second-highest in the European region (24% in women and 22% in men) after the American region (30% in women and 24% in men) [1]. In Sweden, this rate has doubled within the past 10 years [2], and the prevalence of morbid obesity (body mass index (BMI) ≥ 40 kg/m^2^, or BMI ≥ 35 kg/m^2^ plus at least one obesity-related comorbidity) has increased markedly [2, 3]. Obesity is a cause of early mortality, reduced health-related quality of life (HRQoL), and increased risk for cancer, diabetes mellitus, and cardiovascular disorders [4]. Bariatric surgery is the most effective treatment for weight reduction in people living with morbid obesity [5]. This surgery improves obesity related comorbidities including diabetes [6] and cardiovascular disease [7]. A steady increase in the utilization of bariatric surgery has been observed worldwide, with more than 600,000 procedures now being performed annually [8].

Risk prediction models are used to guide clinicians and patients in a joint decision-making process for the selection of appropriate treatments [9]. For bariatric surgery, postsurgical weight [10], remission of associated comorbidities [11], and postsurgical complications [12] are the common predicted outcomes, and risk predictors include patient characteristics (e.g., age, sex, and baseline weight), socioeconomic status, medical history (e.g., comorbidities), and genetic markers. However, as the ultimate goal of bariatric surgery is to improve health, understanding how bariatric surgery might affect HRQoL is important. A few studies have investigated this issue [13–16], and there is a scarcity of studies developing prediction models for HRQoL. The two available prediction models developed by Cao et al. predict HRQoL measured by the Obesity-related Problems Scale and SF-36 (including both sub-domain scores and summary scores) at 2 and 5 year [17, 18], respectively. These models are useful in terms of supporting clinicians and patients in making decisions. However, for resource allocation decision-making within the health sector, it would also be useful to have a single overall measure such as quality-adjusted life years (QALYs).

QALYs are a summary measure that incorporates both life years and quality of life, which enables comparisons across different disease areas and populations. QALYs are required for economic evaluation involving treatment comparisons by many national reimbursement agencies and advisory bodies, such as the National Institute for Health and Clinical Excellence (NICE) in the UK and the Dental and Pharmaceutical Benefits Agency in Sweden [19, 20]. QALYs are calculated by multiplying the duration of time spent in a health state by its health utility. Health utility, which preference for a particular health state, is a numeric value that varies between 0 and 1, where 0 represents “dead” and 1 represents “full health”. Health utility can be estimated by the standard gamble, time trade-off or rating scale methods. However, as these direct valuation methods are often difficult to implement, in practice, a pre-scored preference-based measure (PBM) [21], such as the EQ-5D [22], SF-6D [23], or the health utilities index (HUI) [24], is applied. The psychometric properties of the SF-36 instrument has been estimated among patients with obesity, the validity and reliability of the instrument was accessed with other clinical indicators and disease specific instrument [25, 26]. SF-36 is the most commonly applied generic measure among patients with obesity [25, 26], as well as in Sweden [27]. However, as the SF-36 is not a preference measure, the SF-36 scores cannot be used to access health utility score. The *short form six-dimensions (SF-6D)* was developed to obtain health utility score from the SF-36 [28] or its 12-item version (SF-12), using a *standard gamble* method [29].

There is an association between weight reduction following bariatric surgery and enhancements in HRQOL. However, this may not always be the case due to post-surgery complications or emotional/functional difficulties [30]. The psychological consequences of bariatric surgery need further investigation. Previous studies have mainly reported on HRQOL for up to one-year post-surgery, with only a few studies reporting on longer-term follow-ups (≥ 2 years) [14], It has been found that HRQOL is more likely to improve within the first two years post-surgery, but further investigations is needed to fully understand HRQoL in the longer term. The study aimed to investigate whether QALYs of the patients who underwent bariatric surgery could be predicted using baseline information.

## Methods

### Data sources

Data of all patients who received bariatric surgery in Sweden between January 1, 2011 and March 31, 2019 (n = 47,653) were obtained from the Scandinavian Obesity Surgery Registry (SOReg) for the current study. SOReg is a national quality registry for bariatric surgery in Sweden with a coverage of > 98% nationwide [31]. Information including patients’ sociodemographic characteristics, details regarding the procedure and postsurgical conditions, HRQoL assessed by the RAND-36 and Obesity-related Problems Score. HRQoL data were reported by the patients prior to surgery and at 1, 2, and 5 years postoperatively. Due to the large proportion of missing data (> 60%), QALYs at 5 years were not analyzed in the current study. Ethical permission for analyzing the data was granted by the Ethics Authority in Sweden (reference number: 2019–03666).

### Health outcomes

The SF-36 measures HRQoL in eight domains (social functioning, physical function, role-physical, bodily pain, general health, vitality, social functioning, role-emotional, and mental health), and the SF-36-v1 has been applied in the SOReg [15, 21]. The short form six-dimensions (SF-6D) was developed to derive a preference-based score from the SF-36 [28] or its 12-item version (SF-12) [29]. A brief description of the method was provided below: firstly, 11 items from the original SF-36 were selected to construct the SF-6D six domains, which including pain, mental health, physical functioning, social functioning, role limitations, and vitality, with each domain described on 4–6 functional levels. The combination of the different domains and severity levels defines in total 18 000 health profiles. Step 2, a subset of 249 health profiles were selected, and valued by a representative sample from the general population, using standard gamble (SG) method. Step 3, various econometric models were tested, to estimate a social value set. Such value sets have been established in several countries, in the current study, the UK tariff were applied [28] since no local Swedish tariff was available.

QALYs were calculated using the area under the curve (AUC) method: the SF-6D index at each time point was represented as data points, which were first connected by straight lines to define the “curve”. Then, the AUC was calculated by adding the areas under the curve between each pair of consecutive observations [32].

### Predictors

The following variables were included as predictors in the current study:

Baseline demographic and health characteristics (including age, sex, height, weight, BMI, waist circumference, HbA1c, education level, and smoking status), year of operation, operation time (in minutes), postoperative care period (in days), comorbidities (including sleep apnea, hypertension, diabetes, dyslipidemia, dyspepsia, diarrhea, depression (defined as pharmacological treatment for depression in the present study), and other illnesses), history of deep vein thrombosis (DVT) or pulmonary embolism (PE), previously cholecystectomy, previously anti-reflux surgery, surgery type, primary surgery, initially planned two-step operation, surgical access, reason for conversion, operation method, other simultaneous surgery (including cholecystectomy, gynecological surgery, incisional hernia or umbilical hernia repair, splenectomy, clearing of adhesions more than 10 min, cruroplasty, and other surgeries), specific intra-operative complication (including bleeding, leakage, abscess/deep infection, wound rupture, other wound complications, bowel obstruction/ileus, band-related complications, port-related complications, stricture, marginal ulcer, cardiovascular complication, DVT/PE, pulmonary complication, urinary tract infection, other complications), severity of complication, and baseline operation score and the 11 SF-36 items for constructing SF-6D (including three items for physical function, one for role-physical, two for bodily pain, one for vitality, one for social functioning, one for role-emotional, and two for mental health).

Binary and multi-nominal variables were converted into one or multiple dummy variables, and continuous and ordered variables were standardized to have a mean of 0 and a standard deviation of 1. HbA1c was log transformed before standardization due to its asymmetric distribution.

Missing values in the predictors were imputed using the multiple imputation method in R using the “mice” package [33]. This involved predictive mean matching with chained equations and k-nearest neighbors to draw imputed values, and five imputed datasets were generated for the later prediction analysis [34].

### Prediction models

A general linear regression model and regularized linear regression models, including Lasso regression (with L1 regulation), ridge regression (with L2 regulation), and elastic net regression (with L1 and L2 regulations together) [35] were used to predict postoperative QALYs at follow-up years 1 and 2.

A recursive variable elimination (RVE) method was used during model training, which selected variables by recursively considering smaller and smaller sets of variables [36]. The models were first trained on the initial set of variables, then the importance of each variable was evaluated, and lastly the least important variables were pruned from the current set of variables. The procedure was recursively repeated on the pruned set until the desired number of variables that maximized the R^2^ was eventually reached. The desired optimal number of variables were arbitrarily set to 1, 11, 21, 31, 41, 51, 61, and 71 in the current study. For hyperparameter tuning during the training of the Lasso regression, ridge regression, and elastic net regression models, the exhaustive grid search method over the penalty parameter λ and L1 penalty ratio to maximize the R^2^ was used to determine the optimal parameters [37]. The relative importance of the variables selected in each model was evaluated used the permutation importance [38].

### Model training, validation and test

The whole dataset was randomly split into two parts: a training dataset with 80% of the patients and an external test dataset with 20% of the patients. For the training dataset, k-fold (k = 5 in the current study) cross-validation was used. This approach involved randomly dividing the training data into k groups of equal size. Afterwards, the models were trained on the k-1 folds, and the remaining one fold was used to validate the models. The process was repeated until the models were validated on all the k folds. For each patient in the training dataset, the predicted value was that he/she obtained during in the validation. The final models were then evaluated using the test dataset [39].

### Model performance metrics

The performance of the models was evaluated using R^2^ and relative root mean squared error (RRMSE) [40]. R^2^ is the percentage of the variation in the outcome variable that is explained by the predictors. It is commonly used to check how well-observed results are reproduced by linear models, depending on the proportion of total deviation of results explained by the models. Although adjusted R^2^ is recommended for comparing performance between models, there was no detectable difference between the R^2^ and adjusted R^2^ due to the considerable number of variables included in the final models [41]. RRMSE is another common way of measuring the performance of the prediction in statistical modelling, particularly regression analyses. RRMSE quantifies deviations from the true values relative to the mean of the true values. The smaller the RRMSE is, the better. A model is considered excellent when RRMSE ≤ 10%, good if 10% < RRMSE ≤ 20%, fair if 20% < RRMSE ≤ 30%, and poor if RRMSE > 30% [40].

Because five test datasets were used for prediction purposes, the final R^2^ and RRMSE of a model were calculated as the average values of those from the five datasets.

All analyses were conducted in Python 3.7 (Python Software Foundation, Beaverton, OR, U.S.) and R 4.1.1 (R Foundation for Statistical Computing, Vienna, Austria).

## Results

### Patient characteristics

After excluding patients with age < 18 years, BMI < 30 kg/m^2^, HbA1c < 10 mmol/mol, postoperative care period > 365 days, and incomplete HRQoL form, 32,232 and 16.141 patients were included in the final analysis for follow-up years 1 and 2, respectively. The average age of the patients was 41.2 years, with a standard deviation (SD) of 11.3 years, and most were women (76.6%, Table 1). The standardized differences (SDDs) between all the patients and the patients included in the study at follow-up years 1 or 2 were less than 0.1, except for operation time (Table 1 and supplementary Table S1). In general, the included patients were a representative sample of those who received bariatric surgery in Sweden.

**Table 1:** Baseline demographic characteristics and SF-36 item scores of the patients (follow-up year 1)

Variable		All	Excluded	Included	SDD
N (%)		46,753 (100.0)	14,521 (31.1)	32,232 (68.9)	
Age (mean (SD))		41.21 (11.33)	42.92 (11.16)	40.44 (11.31)	0.068
Sex (%)	Female	35,820 (76.6)	11,271 (77.6)	24,549 (76.2)	0.012
	Male	10,933 (23.4)	3250 (22.4)	7683 (23.8)	
Height, cm (mean (SD))		168.92 (8.99)	168.85 (8.87)	168.95 (9.05)	-0.003
Weight, kg (mean (SD))		119.08 (21.38)	119.23 (20.90)	119.02 (21.60)	0.002
BMI, kg/m^2^ (mean (SD))		41.58 (5.63)	41.67 (5.45)	41.54 (5.71)	0.007
Belly circumference, cm (mean (SD))		124.53 (13.97)	124.57 (13.57)	124.51 (14.15)	0.001
HbA1c, mmol/mol (median [IQR])		37 [34, 41]	38 [35, 42]	37 [34, 41]	0.001
Smoking (%)	Yes	4765 (10.2)	1340 (9.2)	3425 (10.6)	0.047
	No	28,781 (61.6)	8663 (59.7)	20,118 (62.4)	
	Occasionally	862 (1.8)	141 (1.0)	721 (2.2)	
	Quitted recently	7213 (15.4)	2620 (18.0)	4593 (14.2)	
	Unknown	5130 (11.0)	1757 (12.1)	3373 (10.5)	
Education (%)	Missing	28,104 (60.1)	7353 (50.6)	20,751 (64.4)	0.088
	9–12 years	9985 (21.4)	3836 (26.4)	6149 (19.1)	
	< 9 years	2141 (4.6)	850 (5.9)	1291 (4.0)	
	> 12 years	6523 (14.0)	2482 (17.1)	4041 (12.5)	
Sleep apnea (%)		4773 (10.2)	1602 (11.0)	3171 (9.8)	0.015
Hypertension (%)		11,865 (25.4)	4128 (28.4)	7737 (24.0)	0.040
Diabetes (%)		6152 (13.2)	2024 (13.9)	4128 (12.8)	0.015
Dyslipidemia (%)		4559 (9.8)	1570 (10.8)	2989 (9.3)	0.027
Dyspepsia (%)		5010 (10.7)	1422 (9.8)	3588 (11.1)	0.021
Diarrhea (%)		726 (1.6)	202 (1.4)	524 (1.6)	0.023
Depression (%)		7481 (16.0)	2057 (14.2)	5424 (16.8)	0.030
Other illness (%)		5017 (10.7)	1347 (9.3)	3670 (11.4)	0.033
PF1 (%)	1	31,202 (66.7)	9757 (67.2)	21,445 (66.5)	0.005
	2	13,566 (29.0)	4177 (28.8)	9389 (29.1)	
	3	1985 (4.2)	587 (4.0)	1398 (4.3)	
PF2 (%)	1	5743 (12.3)	1556 (10.7)	4187 (13.0)	0.024
	2	26,101 (55.8)	8082 (55.7)	18,019 (55.9)	
	3	14,909 (31.9)	4883 (33.6)	10,026 (31.1)	
PF10 (%)	1	3581 (7.7)	832 (5.7)	2749 (8.5)	0.038
	2	16,276 (34.8)	4848 (33.4)	11,428 (35.5)	
	3	26,896 (57.5)	8841 (60.9)	18,055 (56.0)	
RP3 (%)	1	21,503 (46.0)	6034 (41.6)	15,469 (48.0)	0.040
	2	25,250 (54.0)	8487 (58.4)	16,763 (52.0)	
RE2 (%)	1	17,845 (38.2)	4697 (32.3)	13,148 (40.8)	0.036
	2	28,908 (61.8)	9824 (67.7)	19,084 (59.2)	
SF2 (%)	1	1799 (3.8)	365 (2.5)	1434 (4.4)	0.071
	2	5363 (11.5)	1283 (8.8)	4080 (12.7)	
	3	10,281 (22.0)	2861 (19.7)	7420 (23.0)	
	4	11,000 (23.5)	3348 (23.1)	7652 (23.7)	
	5	18,310 (39.2)	6664 (45.9)	11,646 (36.1)	
BP1 (%)	1	6725 (14.4)	2229 (15.4)	4496 (13.9)	0.033
	2	5687 (12.2)	1926 (13.3)	3761 (11.7)	
	3	7081 (15.1)	2211 (15.2)	4870 (15.1)	
	4	16,595 (35.5)	5210 (35.9)	11,385 (35.3)	
	5	8434 (18.0)	2413 (16.6)	6021 (18.7)	
	6	2231 (4.8)	532 (3.7)	1699 (5.3)	
BP2 (%)	1	12,134 (26.0)	4145 (28.5)	7989 (24.8)	0.041
	2	11,295 (24.2)	3693 (25.4)	7602 (23.6)	
	3	12,221 (26.1)	3691 (25.4)	8530 (26.5)	
	4	8161 (17.5)	2293 (15.8)	5868 (18.2)	
	5	2942 (6.3)	699 (4.8)	2243 (7.0)	
MH1 (%)	1	947 (2.0)	205 (1.4)	742 (2.3)	0.056
	2	2136 (4.6)	478 (3.3)	1658 (5.1)	
	3	4423 (9.5)	1116 (7.7)	3307 (10.3)	
	4	7360 (15.7)	2117 (14.6)	5243 (16.3)	
	5	13,607 (29.1)	4225 (29.1)	9382 (29.1)	
	6	18,280 (39.1)	6380 (43.9)	11,900 (36.9)	
MH4 (%)	1	952 (2.0)	192 (1.3)	760 (2.4)	0.060
	2	2434 (5.2)	522 (3.6)	1912 (5.9)	
	3	4278 (9.2)	1086 (7.5)	3192 (9.9)	
	4	7237 (15.5)	2054 (14.1)	5183 (16.1)	
	5	15,424 (33.0)	4864 (33.5)	10,560 (32.8)	
	6	16,428 (35.1)	5803 (40.0)	10,625 (33.0)	
VT2 (%)	1	1023 (2.2)	352 (2.4)	671 (2.1)	0.047
	2	4248 (9.1)	1532 (10.6)	2716 (8.4)	
	3	7103 (15.2)	2391 (16.5)	4712 (14.6)	
	4	11,120 (23.8)	3702 (25.5)	7418 (23.0)	
	5	13,868 (29.7)	4093 (28.2)	9775 (30.3)	
	6	9391 (20.1)	2451 (16.9)	6940 (21.5)	
Operation score (median [IQR])		70.8 [50.0, 83.3]	66.7 [45.8, 83.3]	70.8 [50.0, 87.5]	0.053
Operation time, minutes (median [IQR])		58 [43, 43]	65 [50, 86]	55 [40, 40]	0.132
Postoperative care period, days (median [IQR])		1 [1, 2]	2 [1, 2]	1 [1, 2]	0.001
QALYs at 1 year (mean (SD))		–	–	0.72 (0.10)	–

Student’s t test or the Mann‒Whitney U test were used for continuous variables, and the chi-squared test was used for categorical variables.

BP, bodily pain; MH, mental health; PF, physical function; RE, role-emotional; RP, role participation; SF, social function; VT, vitality.

SDD: standardized difference

Similarly, although statistically significant differences were found in the SF-36 item scores, in general, the differences were not of clinical significance. Nevertheless, the inverse probability weighting (IPW) method was used in later analyses to account for the probability of a patient being included in the prediction.

The mean QALYs of the included patients at follow-up years 1 and 2 were 0.72 (SD = 0.10) and 1.50 (SD = 0.20), respectively (Tables 1 and S1).

### Model performance

**Fig. 1:**
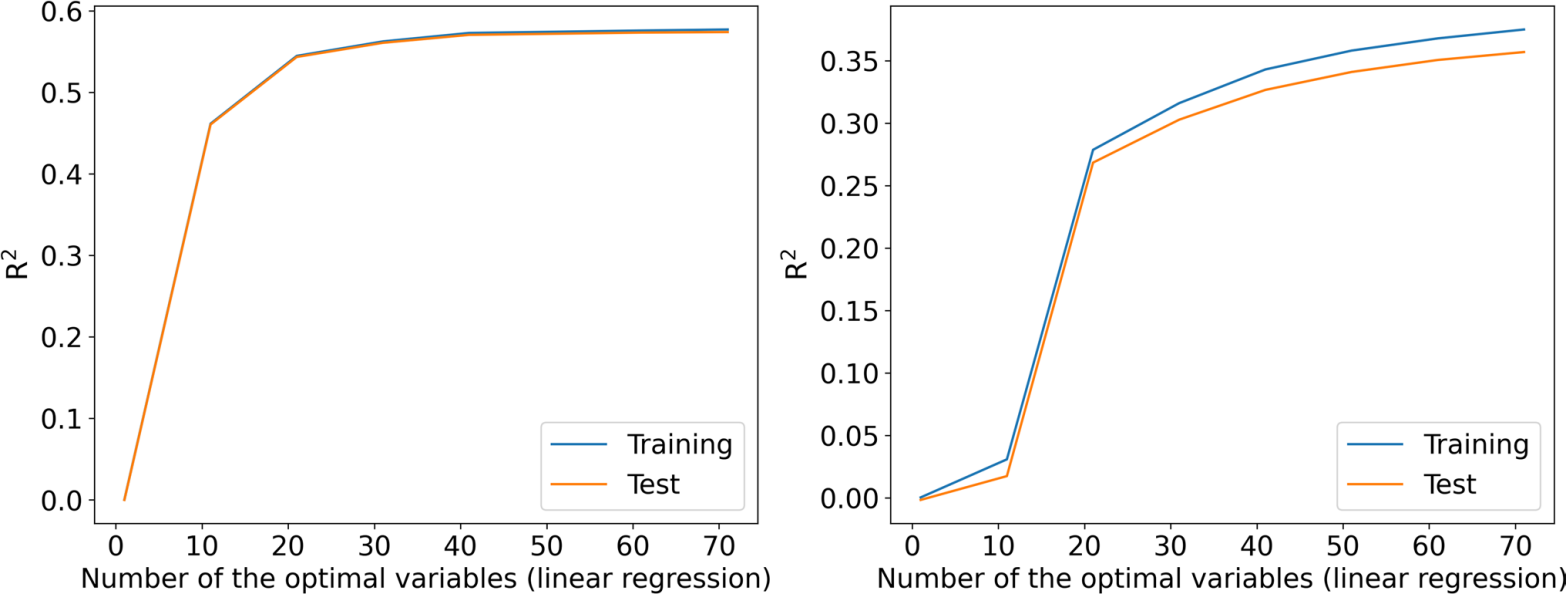
R^2^ of the general linear regression by number of the optimal variables (left, follow-up year 1; right, follow-up year 2)

**Fig. 2:**
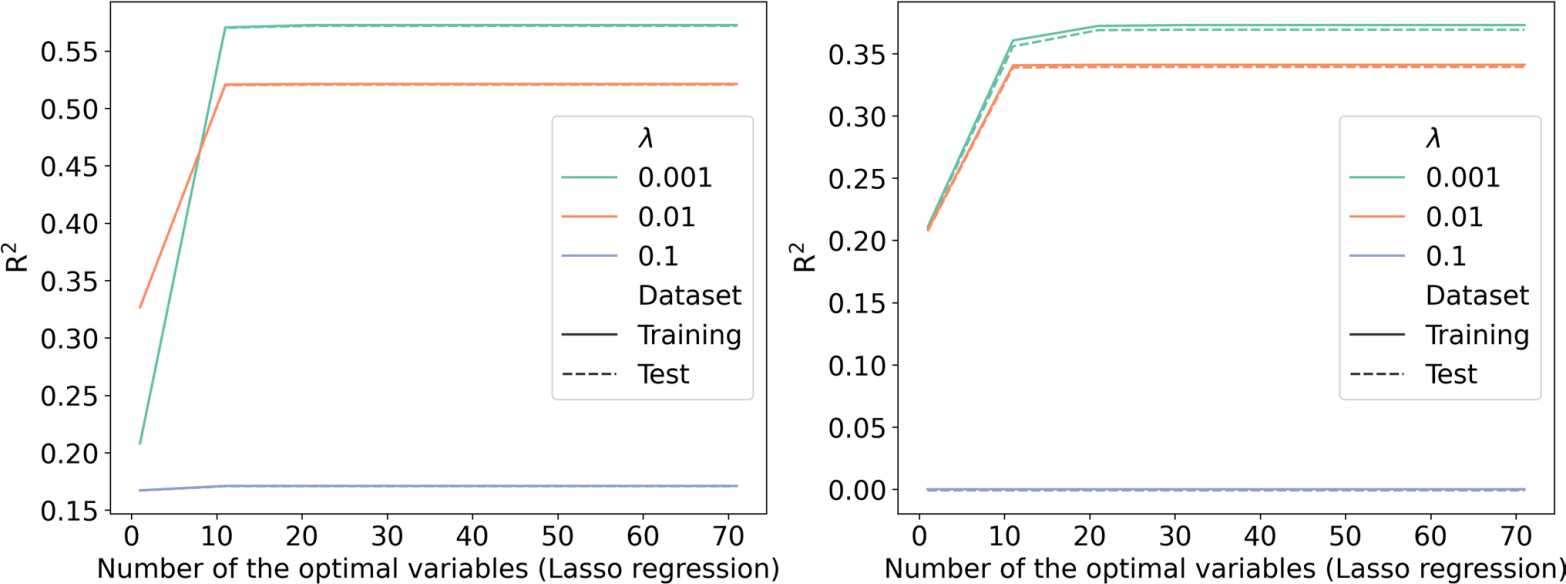
R^2^ of the Lasso regression by number of the optimal variables and penalty parameter (left, follow-up year 1; right, follow-up year 2)

**Fig. 3:**
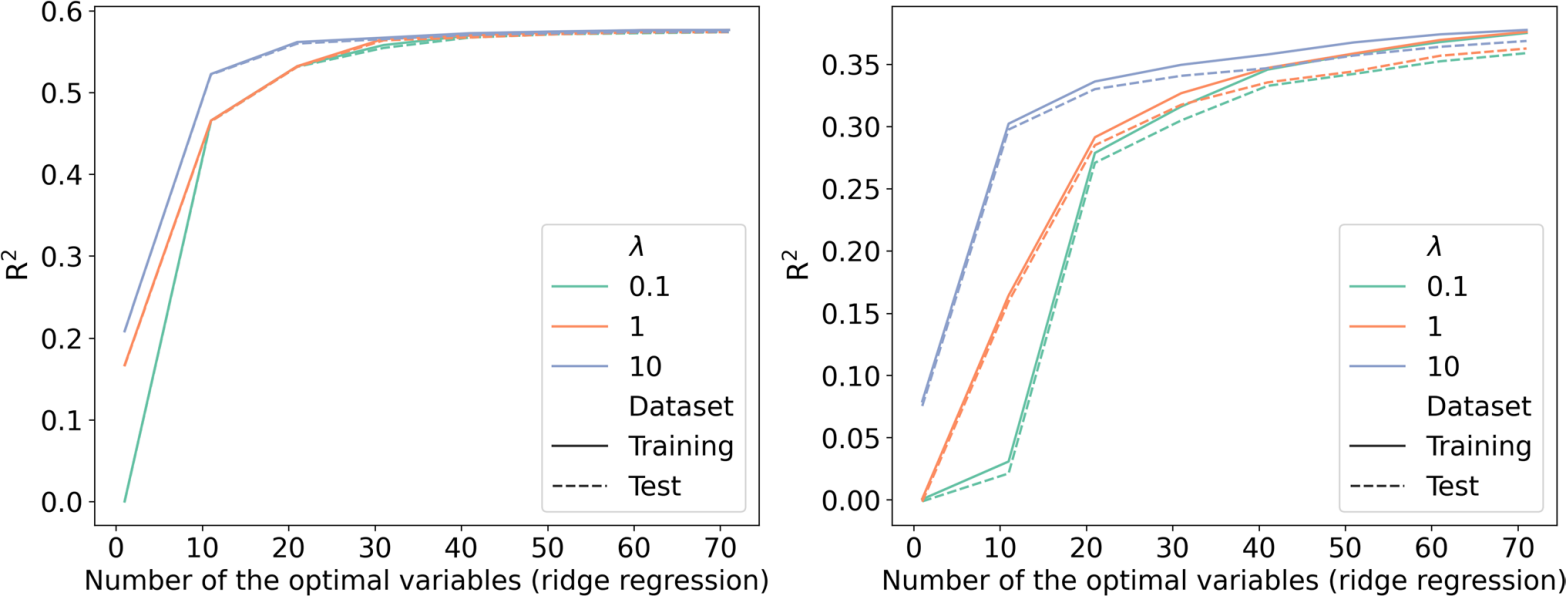
R^2^ of the ridge regression by number of the optimal variables and penalty parameter (left, follow-up year 1; right, follow-up year 2)

**Fig. 4:**
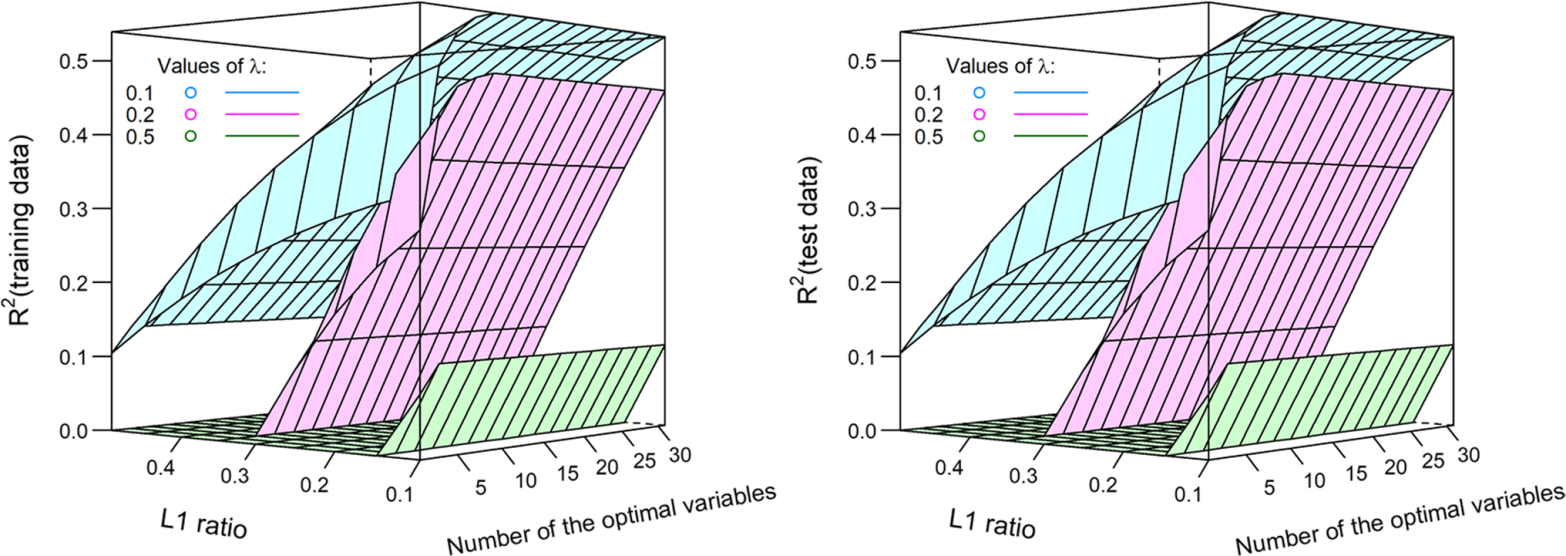
R^2^ of the elastic net regression for follow-up year 1 by number of optimal variables, penalty parameter, and L1 penalty ratio (left: training data; right: test data)

**Fig. 5:**
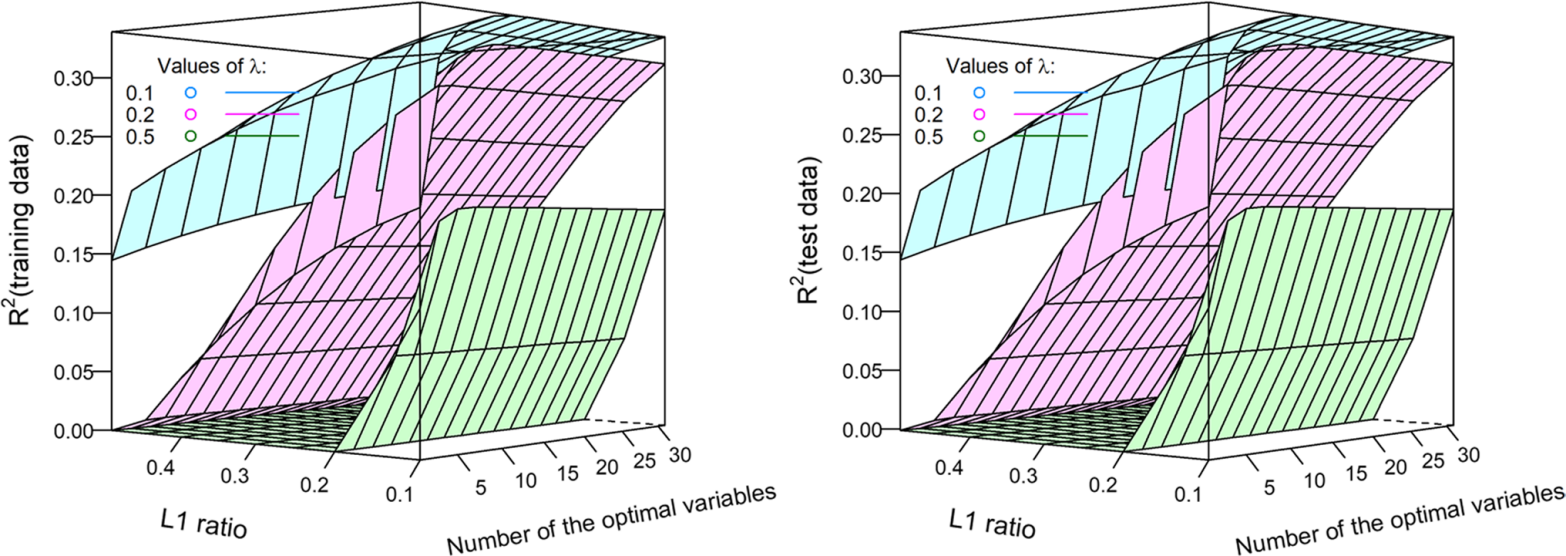
R^2^ of the elastic net regression for follow-up year 2 by number of optimal variables, penalty parameter, and L1 penalty ratio (left: training data; right: test data)

In general, all the models showed equivalent performance by means of R^2^ for both training and test data, without any observed overfitting (Figs. 1–5). For the general linear regression model, the performance of the model increased with the number of variables; however, the improvement was ignorable when the number of variables was more than 30 and 50 for follow-up years 1 and 2, respectively (Fig. 1).

The results indicated that L1 regularization could not improve model performance because R^2^ decreased with the larger penalty parameter λ used in the larger Lasso regression model (Fig. 2).

Although L2 regularization may improve the model performance, indicated by the increased R^2^ with a larger penalty parameter λ, the improvement was negligible when the number of variables was more than 30 and 40 for follow-up years 1 and 2, respectively (Fig. 3).

The elastic net regression models showed similar results: less L1 regularization and a smaller L1 penalty parameter λ corresponded to a larger R2 (Figs. 4 and 5). This meant that less regularization presented better prediction ability. When more variables were included, the performance improved. However, the improvement was negligible when the number of variables was more than 20 (Figs. 4 and 5).

The performance metrics of the optimal models are summarized in Table 2. In general, the four models showed equivalent prediction ability for QALYs at follow-up year 1, with an R^2^ of approximately 0.57 and an RRMSE of approximately 9.6%. The results indicated that the linear regression model, whether regularized or not, may explain about 60% of the variance of 1-year QALYs after bariatric surgery, and the relative error of prediction is less than 10%, suggesting an excellent prediction ability.

**Table 2:** Performance of the models in terms of R^2^ and RRMSE

Model	Follow-up year	R^2^	RRMSE (%)	Variables selected
General linear regression	1	0.573	9.58	Male, smoking, education, ongoing treatment, surgery type, non-laparoscopy, reason of convert, non-GBP, previous anti-reflux surgery, primary surgery, step 1 in a duodenal switch operation, other simultaneous surgery, postoperative complications within 6-weeks, intra-operative complications, bleeding amount, severity of postoperative complication, and SF-36 items,
	2	0.344	15.98	Height, weight, BMI, smoking, education, surgery type, non-laparoscopy, reason of convert, non-GBP, operation duration, ongoing treatment, previous anti-reflux surgery, initially planned 2-step operation, step 1 in a duodenal switch operation, other simultaneous surgery, postoperative complications within 6-weeks, intra-operative complications, severity of postoperative complication, and SF-36 items
Lasso regression	1	0.572	9.72	Age, height, weight, BMI, belly circumference, HbA1c, operation duration, postoperative care period, year of operation, depression, annual procedure volume of hospital, and SF-36 items
	2	0.350	15.90	Age, male, height, weight, BMI, belly circumference, HbA1c, smoking, education, surgery type, non-laparoscopy, reason of convert, non-GBP, operation duration, postoperative care period, year of operation, ongoing treatment, DVT or PE, previous cholecystectomy, previous anti-reflux surgery, primary surgery, initially planned 2-step operation, annual procedure volume of hospital, and SF-36 items,
Ridge regression	1	0.574	9.58	Male, smoking, education, surgery type, non-laparoscopy, reason of convert, non-GBP, ongoing treatment, previous anti-reflux surgery, primary surgery, step 1 in a duodenal switch operation, other simultaneous surgery, postoperative complications within 6-weeks, bleed amount, severity of postoperative complication, and SF-36 items, intra-operative
	2	0.350	15.91	Height, weight, BMI, smoking, education, surgery type, non-laparoscopy, reason of convert, non-GBP, operation duration, ongoing treatment, previous anti-reflux surgery, primary surgery, initially planned 2-step operation, step 1 in a duodenal switch operation, other simultaneous surgery, postoperative complications within 6-weeks, intraoperative complication, bleed amount, severity of postoperative complication, and SF-36 items
Elastic net regression	1	0.569	9.58	Bleed amount, severity of postoperative complication within 6-weeks, and SF-36 items
	2	0.347	15.82	Height, weight, BMI, male, belly circumference, HbA1c, operation duration, postoperative care period, year of operation, and SF-36 items

However, all the models showed poorer performance for predicting 2-year QALYs. Although the RRMSEs (approximately 16%) indicated a good performance of the models, the R^2^ of the models was only approximately 35%.

The selected variables for the optimal models are also presented in Table 2. In general, baseline SF-36 items, sex, age, height, weight, ongoing treatment, postoperative complications, and postoperative complications within 6 weeks etc., were the critical variables for predicting postoperative QALYs. The most parsimonious model was the elastic net regression model, which used fewer variables to predict the QALYs at follow-up year 1 while providing almost equivalent performance (Table 2).

The relative importance of the variables selected in each model for follow-up year 1 is shown in the supplementary Figure S1, which indicates that baseline SF-36 items BP2, SF2, VT2, PF10, MH1, MH4, RP3, RE2, and BP1 are relatively more important for the prediction. However, other variables contributed little to the prediction, which was partially due to that each of the multinominal variables was split into several dummy variables.

## Discussion

In the current study, baseline indicators of importance for predicting post bariatric surgery QALYs were identified, including health-related quality of life, age, sex, BMI, smoking, glycemic control, pre- and post-operative complications within 6 weeks, may provide important information to predict postoperative QALYs of the patients. Additionally, factors unrelated to the patient, in particular year of surgery and annual hospital volume are also of importance. The results of the study could be useful for supporting decision-making in health resource allocation. The QALYs improved after bariatric surgery both at year 1 and year 2 relative to the baseline, with a slightly higher improvement from year 1 to 2 (1.50–0.72 = 0.78) compared with that from baseline to year 1 (0.72). This finding is in line with previous studies showing that bariatric surgery is effective in improving health among patients with morbid obesity [42].

The current study focused mainly on baseline characteristics and their prediction ability on postsurgical QALYs. The ability of baseline characteristics to predict post-surgical QALYs was generally effective at year 1 but showed a relative decrease at year 2. This indicates that relying solely on baseline characteristics may be useful to predict QALYs related to the initial weight-loss phase, but remains insufficient to predict long-term QALYs for which information on post-surgical weight, complications, behavioral traits and comorbidities may also be necessary [17, 18]. Additionally, other factors such as mental health and social support are likely to play significant roles in patients' HRQoL also [43, 44]. Baseline HRQoL (defined by the SF-36 items) was found to be important for both year 1 and 2’s QALYs across all the models, in line with previous studies [17, 18]. This was also confirmed by the relative importance of the HRQoL variables for the prediction of follow-up year 1 (Figure S1).

Both socioeconomic status and lifestyle factors are known to be strongly associated with health [20]. Specifically, age, sex, BMI, and socioeconomic status have all been associated with HRQoL changes in post bariatric surgery [13, 15]. These associations were confirmed in the present study (Table 2). In addition, smoking was an important predictor for postoperative health, which also concurred with a previous study [13]. Knowledge about these factors can help identify groups in need of more intense and individualized support in the preoperative, perioperative and postoperative settings. However, it remains important to stress that while some factors are associated with lower improvement in perceived health, all groups benefited from the surgical intervention and thus should not be excluded from this important treatment. In addition, suffering from a surgical complication was associated with reduced HRQoL improvement. This finding supports a previous study suggesting that patients who suffer from a serious postoperative complication have a higher use of antidepressants and opioids, as well as a higher rate of readmissions over two years after surgery [45]. On the other hand, preoperative use of antidepressants or chronic opioid use might be associated with increased risk for overall and serious postoperative complications [46, 47]. Increased awareness of the mental impact of a serious postoperative complication and the availability of increased support for patients who suffer from such complications may be important.

Our findings indicated that L1 or L2 regularization might improve the performance of the linear models used to predict the 1-year postoperative QALYs of bariatric patients; however, the improvement was minor. Twenty would be a proper number of the variables to be used to predict the 1-year QALYs based on the current data available. Although RRMSE is less than 10%, an R^2^ of 60% is moderate, which might be improved with more information at baseline.

### Strengths and limitations

QALYs combine both quality of life (health utility) and quantity of life (life years) simultaneously and can summarize health outcomes from multiple follow-ups, which might provide a better overall measure of health improvement. Information from this study is useful for economic evaluation studies. The variables used in the study were all based on data from high-quality sources that are continuously validated.

However, there are also limitations in the current study. First, QALYs’ weights were based on the UK population, as no Swedish SF-36 tariff is currently available. Therefore, potential deviation in the QALYs estimates for the Swedish patient population might be exist, which needs to be verified in the future. Second, missingness in the current HRQoL data is substantial, especially for follow-up year 2, which is common in HRQoL studies. Although we did not detect a significant difference in baseline characteristics between the whole patient population and the patients who had follow-up information, the assumed missing at random mechanism for multiple imputation might not hold, and potential sampling bias could not be ruled out [48].

## Conclusions

Patient characteristics before bariatric surgery including health-related quality of life, age, sex, BMI, smoking, glycemic control, pre- and post-operative complications within 6 weeks, may provide important information to predict postoperative QALYs of the patients. Additionally, factors unrelated to the patient, in particular year of surgery and annual hospital volume are also of importance. Knowledge of these factors can help identify groups in need of more intense and individualized preoperative, perioperative and postoperative support.

## Supplementary Information

Below is the link to the electronic supplementary material.

Supplementary file1 (DOCX 138 KB)

## Data Availability

Data sharing is not possible according to Swedish law.

## References

[1] World Health Organization Regional office for Europe. WHO European regional obesity report 2022. Copenhagen, Denmark: World Health Organization; 2022.

[2] Neovius M, Janson A, Rossner S (2006). Prevalence of obesity in Sweden. Obes Rev.

[3] Yumuk V, Tsigos C, Fried M, Schindler K, Busetto L, Micic D, Toplak H, Obesity Management Task Force of the European Association for the Study of, O (2015). European Guidelines for Obesity Management in Adults. Obes Facts.

[4] Kolotkin RL, Meter K, Williams GR (2001). Quality of life and obesity. Obes Rev.

[5] Chang SH, Stoll CR, Song J, Varela JE, Eagon CJ, Colditz GA (2014). The effectiveness and risks of bariatric surgery: an updated systematic review and meta-analysis, 2003–2012. JAMA Surg.

[6] Sundbom M, Hedberg J, Marsk R, Boman L, Bylund A, Hedenbro J, Laurenius A, Lundegardh G, Moller P, Olbers T, Ottosson J, Naslund I, Naslund E, Registry SOS (2017). Substantial Decrease in Comorbidity 5 Years After Gastric Bypass A Population-based Study From the Scandinavian Obesity Surgery Registry. Ann Surg.

[7] Strazzullo P, D'Elia L, Cairella G, Garbagnati F, Cappuccio FP, Scalfi L (2010). Excess Body Weight and Incidence of Stroke Meta-Analysis of Prospective Studies With 2 Million Participants. Stroke.

[8] Angrisani L, Santonicola A, Iovino P, Vitiello A, Higa K, Himpens J, Buchwald H, Scopinaro N (2018). IFSO Worldwide Survey 2016: Primary, Endoluminal, and Revisional Procedures. Obes Surg.

[9] Moons KGM, Royston P, Vergouwe Y, et al. Prognosis and prognostic research: What, why, and how? BMJ-Br Med J. 2009;338(7690):b375. 10.1136/bmj.b375 19237405

[10] Albert U, Bonavigo T, Moro O, et al. SCL-90 empirical factors predict post-surgery weight loss in bariatric patients over longer time periods. Eat Weight Disord. 2022;27(7):2845–2855. 10.1007/s40519-022-01424-4 PMC9556354 35829901

[11] Zhang R, Borisenko O, Telegina I, Hargreaves J, Ahmed AR, Sanchez Santos R, Pring C, Funch-Jensen P, Dillemans B, Hedenbro JL (2016). Systematic review of risk prediction models for diabetes after bariatric surgery. Br J Surg.

[12] Stenberg E, Cao Y, Szabo E, Naslund E, Naslund I, Ottosson J (2018). Risk Prediction Model for Severe Postoperative Complication in Bariatric Surgery. Obes Surg.

[13] Gryth K, Persson C, Naslund I, Sundbom M, Naslund E, Stenberg E (2019). The Influence of Socioeconomic Factors on Quality-of-Life After Laparoscopic Gastric Bypass Surgery. Obes Surg.

[14] Hachem A, Brennan L (2016). Quality of Life Outcomes of Bariatric Surgery: A Systematic Review. Obes Surg.

[15] Raoof M, Szabo E, Karlsson J, Naslund E, Cao Y, Naslund I (2020). Improvements of health-related quality of life 5 years after gastric bypass What is important besides weight loss? A study from Scandinavian Obesity Surgery Register. Surg Obesity Related Dis.

[16] Nielsen HJ, Nedrebo BG, Fossa A, Andersen JR, Assmus J, Dagsland VH, Dankel SN, Gudbrandsen OA, Ferno J, Hjellestad I, Hjermstad MJ, Kolotkin RL, Thorsen HL, Mellgren G, Flolo TN (2022). Seven-year trajectories of body weight, quality of life and comorbidities following Roux-en-Y gastric bypass and sleeve gastrectomy. Int J Obes (Lond).

[17] Cao Y, Raoof M, Montgomery S, et al. Predicting long-term health-related quality of life after bariatric surgery using a conventional neural network: A study based on the Scandinavian obesity surgery registry. J Clin Med. 2019;8(12):2149. 10.3390/jcm8122149 PMC6947423 31817385

[18] Cao Y, Raoof M, Szabo E, et al. Using bayesian networks to predict long-term health-related quality of life and comorbidity after bariatric surgery: A study based on the Scandinavian obesity surgery registry. J Clin Med. 2020;9(6):1895. 10.3390/jcm9061895 PMC7356516 32560424

[19] Drummond MF, Sculpher MJ, Claxton K, et al. Methods for the economic evaluation of health care programmes: Oxford university press. 2015.

[20] Fayers PM, Machin D (2006). Quality of life: the assessment, analysis and interpretation of patient-reported outcomes.

[21] Ware JE, Gandek B (1998). Overview of the SF-36 Health Survey and the International Quality of Life Assessment (IQOLA) Project. J Clin Epidemiol.

[22] Devlin NJ, Brooks R (2017). EQ-5D and the EuroQol Group: Past, Present and Future. Appl Health Econ Health Policy.

[23] Whitehurst DG, Bryan S, Lewis M (2011). Systematic review and empirical comparison of contemporaneous EQ-5D and SF-6D group mean scores. Med Decis Making.

[24] Horsman J, Furlong W, Feeny D, Torrance G (2003). The Health Utilities Index (HUI): concepts, measurement properties and applications. Health Qual Life Outcomes.

[25] Lindekilde N, Gladstone BP, Lubeck M, Nielsen J, Clausen L, Vach W, Jones A (2015). The impact of bariatric surgery on quality of life: a systematic review and meta-analysis. Obes Rev.

[26] Al Amer R, Al Khalifa K, Alajlan SA, Al Ansari A (2018). Analyzing the Psychometric Properties of the Short Form-36 Quality of Life Questionnaire in Patients with Obesity. Obes Surg.

[27] Karlsen TI, Tveita EK, Natvig GK, Tonstad S, Hjelmesaeth J (2011). Validity of the SF-36 in patients with morbid obesity. Obes Facts.

[28] Brazier J, Roberts J, Deverill M (2002). The estimation of a preference-based measure of health from the SF-36. J Health Econ.

[29] Brazier JE, Roberts J (2004). The estimation of a preference-based measure of health from the SF-12. Med Care.

[30] Groven KS, Raheim M, Engelsrud G (2013). Dis-appearance and dys-appearance anew: living with excess skin and intestinal changes following weight loss surgery. Med Health Care Philos.

[31] Sundbom M, Naslund I, Naslund E, Ottosson J (2021). High acquisition rate and internal validity in the Scandinavian Obesity Surgery Registry. Surg Obes Relat Dis.

[32] Fayers PM, Machin D. Quality of life: The assessment, analysis and interpretation of patient-reported outcomes: UK: John Wiley & Sons. 2013.

[33] van Buuren S, Groothuis-Oudshoorn K (2011). mice: Multivariate Imputation by Chained Equations in R. J Stat Softw.

[34] Royston P (2004). Multiple imputation of missing values. Stata Journal.

[35] Bisong E. Building machine learning and deep learning models on Google cloud platform. Berkeley, CA: Apress; 2019.

[36] Stracuzzi DJ, Utgoff PE (2004). Randomized variable elimination. The J Machine Lear Res.

[37] Kadam VJ, Jadhav SM (2020). Performance analysis of hyperparameter optimization methods for ensemble learning with small and medium sized medical datasets. Journal of Discrete Mathematical Sciences and Cryptography.

[38] Altmann A, Tolosi L, Sander O, Lengauer T (2010). Permutation importance: a corrected feature importance measure. Bioinformatics.

[39] James G, Witten D, Hastie T, et al. An introduction to statistical learning (Vol. 112): Springer. 2013.

[40] Despotovic M, Nedic V, Despotovic D, Cvetanovic S (2016). Evaluation of empirical models for predicting monthly mean horizontal diffuse solar radiation. Renew Sustain Energy Rev.

[41] Helland IS (1987). On the Interpretation and Use of R2 in Regression-Analysis. Biometrics.

[42] Major P, Matlok M, Pedziwiatr M, Migaczewski M, Budzynski P, Stanek M, Kisielewski M, Natkaniec M, Budzynski A (2015). Quality of Life After Bariatric Surgery. Obes Surg.

[43] van Hout GC, Boekestein P, Fortuin FA, Pelle AJ, van Heck GL (2006). Psychosocial functioning following bariatric surgery. Obes Surg.

[44] van Hout G (2005). Psychosocial effects of bariatric surgery. Acta Chir Belg.

[45] Vidarsson B, Lofling Skogar M, Sundbom M (2021). Impact of a severe complication two years after laparoscopic Roux-en-Y gastric bypass: a cohort study from the Scandinavian Obesity Surgery Registry. Surg Obes Relat Dis.

[46] Hah JM, Bateman BT, Ratliff J, Curtin C, Sun E (2017). Chronic Opioid Use After Surgery: Implications for Perioperative Management in the Face of the Opioid Epidemic. Anesth Analg.

[47] Skogar ML, Sundbom M (2021). Preoperative chronic opioid use and its impact on early complications in bariatric surgery: a Swedish nationwide cohort study of 56,183 patients. Surg Obes Relat Dis.

[48] Sun S, Luo N, Stenberg E, et al. Sequential multiple imputation for real-world health-related quality of life missing data after bariatric surgery. Int J Environ Res Public Health. 2022;19(17):10827. 10.3390/ijerph191710827 PMC9518315 36078543

