# Supplementary material for: Prediction of quality-adjusted life years (QALYs) after bariatric surgery using regularized linear regression models: results from a Swedish nationwide quality register
Source: Obes Surg. 2023 Jun 15;33(8):2452–62. doi: 10.1007/s11695-023-06685-1 (PMC10345068; doi:10.1007/s11695-023-06685-1)
Supplement: Supplementary file 1 — Supplementary file1 (DOCX 138 KB) [file 11695_2023_6685_MOESM1_ESM.docx]

Table S1. Baseline demographic characteristics and SF-36 items’ scores of the patients (follow-up year 2)

| Variable |  | All | Excluded | Included | SSD |
| --- | --- | --- | --- | --- | --- |
| N (%) |  | 46753 (100.0) | 30612 (64.5) | 16141 (35.5) |  |
| Age (mean (SD)) |  | 41.21 (11.33) | 41.38 (11.35) | 40.89 (11.27) | 0.028 |
| Sex (%) | Female | 35820 (76.6) | 23601 (77.1) | 12219 (75.7) | 0.022 |
|  | Male | 10933 (23.4) | 7011 (22.9) | 3922 (24.3) |  |
| Height, cm (mean (SD)) |  | 168.92 (8.99) | 168.82 (8.98) | 169.09 (9.02) | -0.019 |
| Weight, kg (mean (SD)) |  | 119.08 (21.38) | 118.76 (21.40) | 119.70 (21.33) | -0.029 |
| BMI, kg/m^2^ (mean (SD)) |  | 41.58 (5.63) | 41.51 (5.66) | 41.71 (5.57) | -0.023 |
| Belly circumference, cm (mean (SD)) |  | 124.53 (13.97) | 124.65 (13.96) | 124.30 (13.99) | 0.016 |
| HbA1c, mmol/mol (median [IQR]) |  | 37.00 [34.00, 41.00] | 37.00 [34.00, 41.60] | 37.00 [34.00, 41.00] | 0.001 |
| Smoking (%) | Yes | 4765 (10.2) | 3014 ( 9.8) | 1751 (10.8) | 0.057 |
|  | No | 28781 (61.6) | 18577 (60.7) | 10204 (63.2) |  |
|  | Unknown | 5130 (11.0) | 3578 (11.7) | 1552 ( 9.6) |  |
|  | Quitted recently | 7213 (15.4) | 4872 (15.9) | 2341 (14.5) |  |
|  | Occasionally | 862 ( 1.8) | 569 ( 1.9) | 293 ( 1.8) |  |
| Education (%) | Missing | 28104 (60.1) | 17788 (58.1) | 10316 (63.9) | 0.082 |
|  | 9-12 years | 9985 (21.4) | 6868 (22.4) | 3117 (19.3) |  |
|  | < 9 years | 2141 ( 4.6) | 1540 ( 5.0) | 601 ( 3.7) |  |
|  | > 12 years | 6523 (14.0) | 4416 (14.4) | 2107 (13.1) |  |
| Sleep apnea (%) |  | 4773 (10.2) | 3137 (10.2) | 1636 (10.1) | 0.004 |
| Hypertension (%) |  | 11865 (25.4) | 7859 (25.7) | 4006 (24.8) | 0.013 |
| Diabetes (%) |  | 6152 (13.2) | 3982 (13.0) | 2170 (13.4) | 0.011 |
| Dyslipidemia (%) |  | 4559 ( 9.8) | 2928 ( 9.6) | 1631 (10.1) | 0.017 |
| Dyspepsia (%) |  | 5010 (10.7) | 3097 (10.1) | 1913 (11.9) | 0.050 |
| Diarrhea (%) |  | 726 ( 1.6) | 442 ( 1.4) | 284 ( 1.8) | 0.056 |
| Depression (%) |  | 7481 (16.0) | 4846 (15.8) | 2635 (16.3) | 0.045 |
| Other illness (%) |  | 5017 (10.7) | 2877 ( 9.4) | 2140 (13.3) | 0.101 |
| PF1 (%) | 1 | 31202 (66.7) | 20304 (66.3) | 10898 (67.5) | 0.017 |
|  | 2 | 13566 (29.0) | 8979 (29.3) | 4587 (28.4) |  |
|  | 3 | 1985 ( 4.2) | 1329 ( 4.3) | 656 ( 4.1) |  |
| PF2 (%) | 1 | 5743 (12.3) | 3633 (11.9) | 2110 (13.1) | 0.033 |
|  | 2 | 26101 (55.8) | 17009 (55.6) | 9092 (56.3) |  |
|  | 3 | 14909 (31.9) | 9970 (32.6) | 4939 (30.6) |  |
| PF10 (%) | 1 | 3581 ( 7.7) | 2310 ( 7.5) | 1271 ( 7.9) | 0.039 |
|  | 2 | 16276 (34.8) | 10380 (33.9) | 5896 (36.5) |  |
|  | 3 | 26896 (57.5) | 17922 (58.5) | 8974 (55.6) |  |
| RP3 (%) | 1 | 21503 (46.0) | 13734 (44.9) | 7769 (48.1) | 0.043 |
|  | 2 | 25250 (54.0) | 16878 (55.1) | 8372 (51.9) |  |
| RE2 (%) | 1 | 17845 (38.2) | 11297 (36.9) | 6548 (40.6) | 0.049 |
|  | 2 | 28908 (61.8) | 19315 (63.1) | 9593 (59.4) |  |
| SF2 (%) | 1 | 1799 ( 3.8) | 1149 ( 3.8) | 650 ( 4.0) | 0.049 |
|  | 2 | 5363 (11.5) | 3379 (11.0) | 1984 (12.3) |  |
|  | 3 | 10281 (22.0) | 6612 (21.6) | 3669 (22.7) |  |
|  | 4 | 11000 (23.5) | 7117 (23.2) | 3883 (24.1) |  |
|  | 5 | 18310 (39.2) | 12355 (40.4) | 5955 (36.9) |  |
| BP1 (%) | 1 | 6725 (14.4) | 4603 (15.0) | 2122 (13.1) | 0.055 |
|  | 2 | 5687 (12.2) | 3863 (12.6) | 1824 (11.3) |  |
|  | 3 | 7081 (15.1) | 4642 (15.2) | 2439 (15.1) |  |
|  | 4 | 16595 (35.5) | 10778 (35.2) | 5817 (36.0) |  |
|  | 5 | 8434 (18.0) | 5353 (17.5) | 3081 (19.1) |  |
|  | 6 | 2231 ( 4.8) | 1373 ( 4.5) | 858 ( 5.3) |  |
| BP2 (%) | 1 | 12134 (26.0) | 8248 (26.9) | 3886 (24.1) | 0.057 |
|  | 2 | 11295 (24.2) | 7492 (24.5) | 3803 (23.6) |  |
|  | 3 | 12221 (26.1) | 7869 (25.7) | 4352 (27.0) |  |
|  | 4 | 8161 (17.5) | 5206 (17.0) | 2955 (18.3) |  |
|  | 5 | 2942 ( 6.3) | 1797 ( 5.9) | 1145 ( 7.1) |  |
| MH1 (%) | 1 | 947 ( 2.0) | 605 ( 2.0) | 342 ( 2.1) | 0.035 |
|  | 2 | 2136 ( 4.6) | 1319 ( 4.3) | 817 ( 5.1) |  |
|  | 3 | 4423 ( 9.5) | 2803 ( 9.2) | 1620 (10.0) |  |
|  | 4 | 7360 (15.7) | 4861 (15.9) | 2499 (15.5) |  |
|  | 5 | 13607 (29.1) | 8868 (29.0) | 4739 (29.4) |  |
|  | 6 | 18280 (39.1) | 12156 (39.7) | 6124 (37.9) |  |
| MH4 (%) | 1 | 952 ( 2.0) | 585 ( 1.9) | 367 ( 2.3) | 0.060 |
|  | 2 | 2434 ( 5.2) | 1446 ( 4.7) | 988 ( 6.1) |  |
|  | 3 | 4278 ( 9.2) | 2675 ( 8.7) | 1603 ( 9.9) |  |
|  | 4 | 7237 (15.5) | 4681 (15.3) | 2556 (15.8) |  |
|  | 5 | 15424 (33.0) | 10155 (33.2) | 5269 (32.6) |  |
|  | 6 | 16428 (35.1) | 11070 (36.2) | 5358 (33.2) |  |
| VT2 (%) | 1 | 1023 ( 2.2) | 706 ( 2.3) | 317 ( 2.0) | 0.056 |
|  | 2 | 4248 ( 9.1) | 2890 ( 9.4) | 1358 ( 8.4) |  |
|  | 3 | 7103 (15.2) | 4735 (15.5) | 2368 (14.7) |  |
|  | 4 | 11120 (23.8) | 7449 (24.3) | 3671 (22.7) |  |
|  | 5 | 13868 (29.7) | 8974 (29.3) | 4894 (30.3) |  |
|  | 6 | 9391 (20.1) | 5858 (19.1) | 3533 (21.9) |  |
| Operation score (median [IQR]) |  | 70.80 [50.00, 83.30] | 70.80 [45.80, 83.30] | 70.80 [50.00, 87.50] | -0.057 |
| Operation time, minutes (median [IQR]) |  | 58.00 [43.00, 78.00] | 60.00 [45.00, 80.00] | 55.00 [39.00, 75.00] | 0.123 |
| Postoperative care period, days (median [IQR]) |  | 1.00 [1.00, 2.00] | 1.00 [1.00, 2.00] | 1.00 [1.00, 2.00] | 0.001 |
| QALYs at 1 year (mean (SD)) |  | - | - | 1.50 (0.20) | - |

Student’s t test or the Mann‒Whitney U test were used for continuous variables, and the chi-squared test was used for categorical variables.

BP, bodily pain; MH, mental health; PF, physical function; RE, role-emotional; RP, role participation; SF, social function; VT, vitality.

SDD: standardized difference


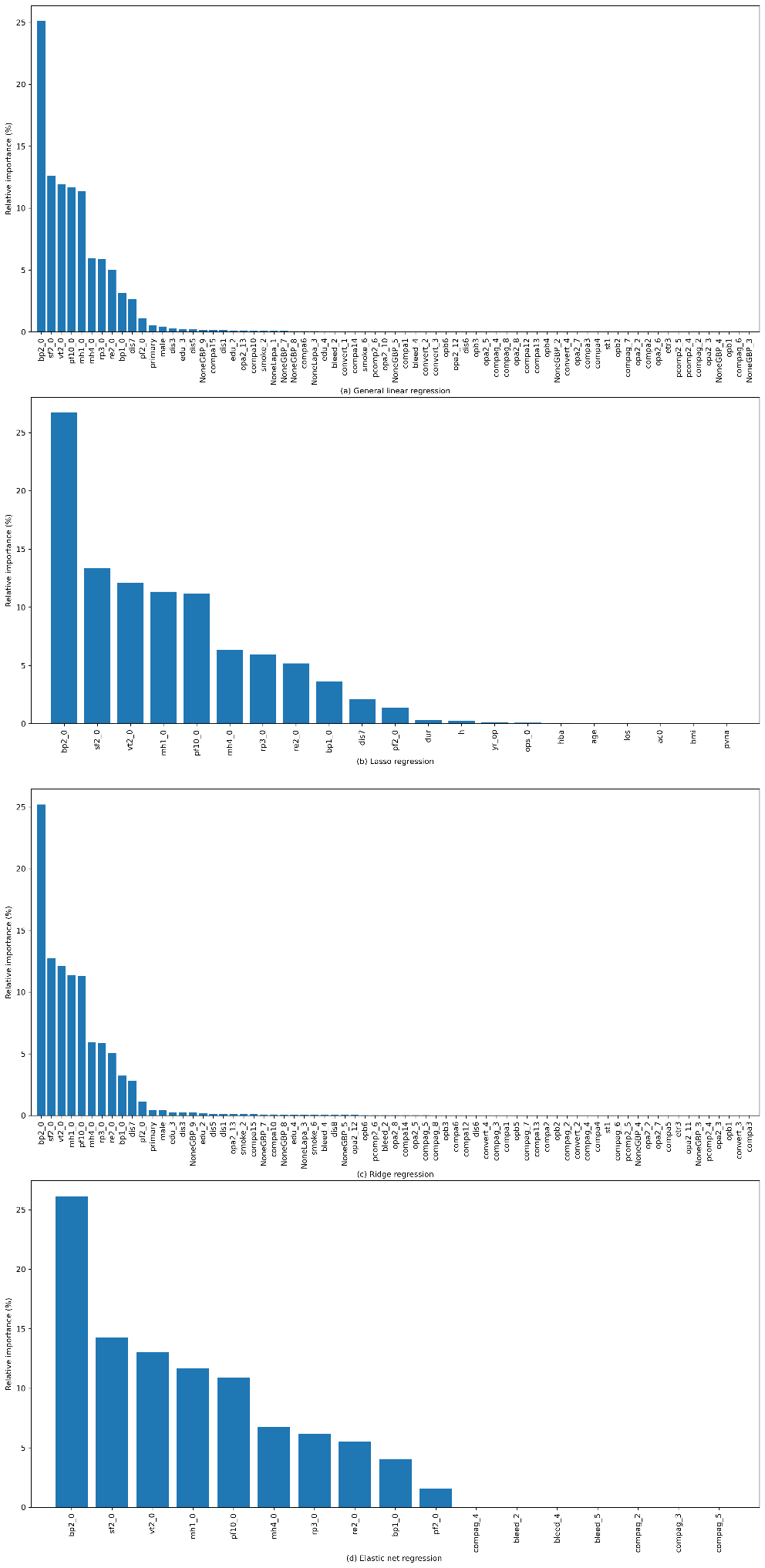


Figure S1. Relative importance of the variables selected in the prediction models
